# The molecular management of classic myeloproliferative neoplasm

**DOI:** 10.1186/s43046-025-00325-8

**Published:** 2025-11-10

**Authors:** Charlène G. S. Soro, Sara Benchikh, Adil El Hamouchi, Imane Morjane, Rachid Saile, Halima Lebrazi, Sanaa Nassereddine

**Affiliations:** 1 Biology and Health Laboratory (LBS) -URAC 34, Faculty of Sciences Ben M’Sik, Hassan II University, Casablanca, Morocco; 2 Cytogenetics Laboratory, Pasteur Institute of Morocco (IPM), 1 Place Louis Pasteur, Casablanca, 20360 Morocco; 3 Laboratory of Human Molecular Genetics, Pasteur Institute of Morocco (IPM), 1 Place Louis Pasteur, Casablanca, 20360 Morocco

**Keywords:** Myeloproliferative neoplasm, Mutation, Molecular analysis, Technique

## Abstract

**Background:**

Whole-genome sequencing has enabled the development of a wide range of analytical tools to search for abnormalities associated with tumors. As classic myeloproliferative neoplasms (MPNs) are associated with genomic alterations in hematopoietic stem cells, the World Health Organization (WHO) recommendations include since 2008 molecular investigations as an important part of the diagnosis and management of these pathologies. Recent advances in sequencing technologies, such as next-generation sequencing (NGS), have enhanced the analysis platforms. However, epidemiological information on MPNs is limited, especially in low/middle-income countries.

**Aim:**

This literature review provides a state-of-the-art on the classification of MPNs and a comprehensive examination of contemporary analytical techniques, while highlighting the advantages and drawbacks of each method.

**Methods:**

The scientific literature for the synthesis of this article was obtained by searching the PubMed and Science Direct databases, and the tables were generated using Excel 2016 software.

**Results:**

Driver mutations in MPNs can be detected by genotyping or sequencing. Genotyping techniques present an increased risk of false negatives because of their low sensitivity, whereas sequencing techniques are more sensitive but can present specificity or time-consuming disadvantages.

**Conclusion:**

Although a large number of applications favor NGS, it is essential to consider the cost-effectiveness of these technologies to meet the needs of laboratories in low/middle-income regions. Alternative techniques such as real-time polymerase chain reaction (qPCR), immunohistochemistry (CAL2IHC), and liquid chromatography (dHPLC) should be explored and considered as sustainable options.

## Introduction

The term myeloproliferative neoplasm (disorders or syndrome) (MPNs) emerged in 1951 with William Dameshek [1] to regroup four diseases with common clinical and biological characteristics: chronic myeloid leukemia (CML), polycythemia vera (PV), essential thrombocythemia (ET), and primary myelofibrosis (PMF). MPNs are acquired malignancies of the myeloid lineage with abnormalities in hematopoietic stem cells (HSC), conferring a proliferative and survival advantage to tumor cells. Since 2008, in addition to the four classic MPNs, other conditions such as chronic neutrophilic leukemia, chronic eosinophilic leukemia, juvenile myelomonocytic leukemia, and unspecified chronic myeloproliferative neoplasm have been added to this group [2, 3]. Genetically, MPNs are clonally heterogeneous and multifactorial group of diseases that differ in their gene expression and clonal architecture. The order of mutation acquisition and germline predisposition can contribute to MPN pathogenesis, as well as differences in the disease phenotype and prognosis [4].

At the molecular level, the common point in MPNs is the untimely activation of the signaling pathway via cytokine receptors or tyrosine kinase adapter mutations. In all cases, the MPN founder variant is a gene involved in cell signaling, epigenetic regulation, or RNA splicing, the latter being particularly common in patients over 70 years of age. Variants of other genes involved in cellular processes are often identified as subsequent events.

In 2005, the JAK2V617F mutation was identified in patients with PV, ET, and PMF [5–7]. Thereafter, mutations in JAK2 exon 12 [8], CALR exon 9 [9], and MPL exon 10 [10] have been detected in a subset of patients most often with ET or PMF. Recently, several studies have identified additional mutations in a small fraction of patient [11–15]. Undoubtedly, it is crucial to gather relevant data for the establishment and follow-up of such diseases. In this context, the choice of method and interpretation of diagnostic evaluations are important.

In low/middle-income countries, the current diagnostic criteria for MPNs are based on anatomopathology, morphology, and cytogenetic or fluorescence in situ hybridization (FISH) findings following the 2016 WHO classification revision [16]. Somatic mutations are rarely incorporated into the diagnostic criteria because of the lack of laboratories that can provide molecular analysis. In this literature review, we discuss the role of molecular biology in the diagnosis of these diseases and the evolution of their technical prognosis. Adaptation of the tool to the diagnostic target is a challenge for biologists and laboratories, as no specific recommendations on the technical level have been formulated by the WHO. Thus, monitoring the tumor burden is crucial for disease monitoring to evaluate the effects of therapies.

**Fig. 1:**
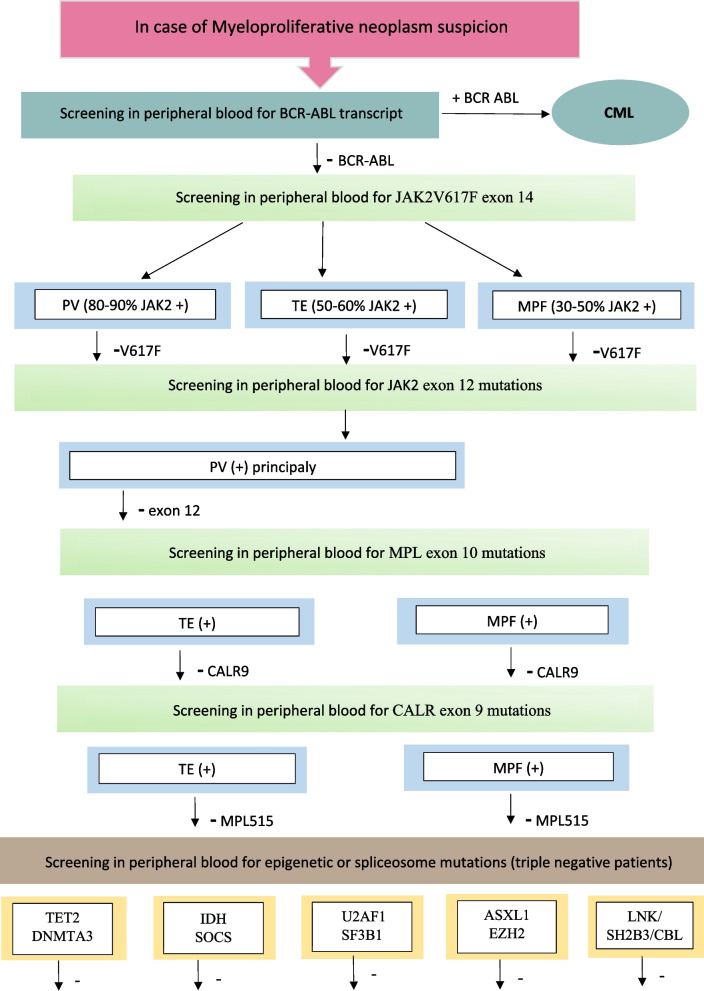
Myeloproliferative neoplasm decision tree

Myeloproliferative neoplasms affect 0.3–1.7/100.000 inhabitants worldwide annually [17]. In Morocco, these pathologies remain poorly understood, despite an overall incidence of new annual cases of MPNs in 2018 estimated to be 2–6% [18, 19]. The objective of this study was to provide a comprehensive overview of MPNs and to update the current state of the art in detecting the most prevalent mutations that affect the development of these disorders. Here, we present a framework (Fig. 1) for making decisions regarding gene detection methods in order to assist practitioners and biologists in the diagnostic process and to improve the current diagnostic system. This framework is beneficial for enhancing the accuracy and efficiency of the diagnostic process. Additionally, enhancing our knowledge of the mutational profiles of patients could provide better epidemiological data in low/middle-income countries and improve patient management.

### The different types of myeloproliferative disorders

Myeloproliferative neoplasms are clonal bone marrow diseases that develop from pluripotent cells. They present with pseudo-similar hematologic symptoms, which have been classified and revised since 2001 by the WHO. Table 1 shows the evolution of the PV, ET, and PMF diagnostic criteria since the 2008 WHO revision. The pathogenesis of MPNs is closely related to the acquisition of specific molecular alterations in stem cells. These disorders present a risk of thrombosis and progression to fibrosis or acute leukemia. The evolution of pathological cells in MPNs can be divided into three phases:

- The precancerous stage initiates the disease by a subtle proliferative advantage of HSCs supplied by a hereditary predisposition or acquired defects,
- The symptomatic MPN phase which is triggered by the occurrence of specific oncogenic mutations leading to increased receptor signaling cytokines, and
- The post-acute MPN phase induced by the accumulation of multiple secondary oncogenic events [20].

**Table 1:** BCR-ABL1 negative MPN diagnosis criteria evolution from the WHO 2008 to 2022 [3, 16, 37]

		WHO 2008	WHO 2016	WHO 2022	
	Major criterion				PV
A1	Hemoglobin (Hb) Hematocrit (Hct) Red cell mass (RCM)	Hb > 17–18.5 g/dL (M) Hb > 15–16.5 g dL (F) At the 99th percentile for age sex and altitude of residence RCM > 25% of the reference value	Hb > 16.5 g/dL (M) Hb > 16 g/dL (F) Hct > 49% (M); Hct > 48% (F) RCM > 25% of the reference value	Hb > 16.5 g/dL (M) Hb > 16.0 g/dL (F) Hct > 49% (M); Hct > 48% (F) RCM > 25% above mean normal predicted value	
A2	Bone marrow biopsy	Age-adjusted hypercellularity with trilineage proliferation; Endogenous erythroid colony formation in vitro	Hypercellularity; Panmyelosis with erythroid and megakaryocytic proliferation (pleomorphic and mature)	Bone marrow biopsy showing age-adjusted hypercellularity with trilineage proliferation (panmyelosis), including prominent erythroid granulocytic, and increase in pleomorphic, mature megakaryocytes without atypia	
A3	Clonal abnormality	EEC of spontaneous growth in culture and/or mutations of JAK2V167F or JAK2 exon 12	JAK2V167F or JAK2 exon 12 mutation	JAK2V617F or JAK2 exon 12 mutation	
	Minor criterion				
B1	Determination of erythropoietin (EPO) serum	EPO serum below the normal value	Subnormal serum EPO	Subnormal serum EPO	
B2	Secondary cause of polycythemia	-	Previous established diagnosis of PV Bone marrow fibrosis of grade 2 or 3	Previous established diagnosis of PV Bone marrow fibrosis of grade 2 or 3	
	Diagnosis:	Diagnosis of PV requires meeting 2 major criteria and 1 minor criteria, or 1 major criteria and the 2 minor criterion	Diagnosis of PV requires meeting all 3 major criteria, or the first 2 major criteria and the minor criterion	Diagnosis of PV requires meeting all 3 major criteria, or the first 2 major criteria and the minor criterion	
	Major criterion				ET
A1	Platelets (Plts)	Plts ≥ 450 × 10^9^/L Hypercellularity Moderate leukocytosis	Plts ≥ 450 × 10^9^/L Hypercellularity Moderate leukocytosis	Plts ≥ 450 × 10^9^/L Hypercellularity Moderate leukocytosis	
A2	Clonal abnormalities	Demonstration of JAK2V617F or other clonal marker, or in the absence of JAK2V617F, no evidence of reactive thrombocytosis	Presence of a mutation in JAK2, CALR, or MPL	Presence of a mutation in JAK2, CALR, or MPL	
A3	Exclusion criteria	Absence of criteria established by the WHO for diagnosing PV, CML, MDS, or other myeloid neoplasms	Absence of criteria established by the WHO for diagnosing PV, CML, MDS, or other myeloid neoplasms	Absence of criteria established by the WHO for diagnosing PV, CML, MDS, or other myeloid neoplasms	
A4	Bone marrow biopsy	Excessive proliferation of the megakaryocytic lineage, mainly with the production of large, mature cells without significant increase in granulopoiesis or erythropoiesis, or excess of immature elements in these two lineages	Especially the proliferation of megakaryocytic lineage with an increase in the number of mature forms large with a hyper-lobed core. No significant increase in granulocytic and erythroid lineages, with or without increased rates of reticulin fiber	Bone marrow biopsy showing proliferation mainly of the megakaryocytic lineage, with increased numbers of enlarged, mature megakaryocytes with hyper-lobulated staghorn-like nuclei. Infrequently dense clusters. No significant increase or left shift in neutrophil granulopoiesis or erythropoiesis; no relevant bone marrow fibrosis. Absence of evidence of reactive thrombocytosis	
	Minor criterion				
B	Platelets	-	Presence of a clonal marker (e.g., abnormal karyotype) or absence of evidence for reactive thrombocytosis	Presence of a clonal marker or absence of evidence of reactive thrombocytosis	
	Diagnosis:	Diagnosis of ET requires meeting all criteria	Diagnosis of ET requires meeting all 4 major criteria, or the first 3 major criteria and the minor criterion	Diagnosis of ET requires meeting all 4 major criteria, or the first 3 major criteria and the minor criterion	
	Major criterion				PMF
A1	Bone marrow biopsy	Megakaryocyte proliferation and atypia accompanied by either reticulin and/or collagen fibrosis; or in absence of reticulin fibrosis, megakaryocyte changes must be accompanied by increased marrow cellularity, granulocytic proliferation, and often decreased erythropoiesis (i.e., pre-fibrotic PMF)	Pre-PMF: megakaryocytic proliferation and atypia, without reticulin fibrosis > grade 1, accompanied by increased age-adjusted bone marrow cellularity, granulocytic proliferation, and often decreased erythropoiesis Overt PMF: megakaryocyte proliferation and atypia accompanied by either reticulin and/or collagen fibrosis (grade 2 or 3)	Pre-fibrotic—PMF: bone marrow biopsy showing megakaryocytic proliferation and atypia), bone marrow fibrosis grade < 2, increased age-adjusted BM cellularity, granulocytic proliferation, and (often) decreased erythropoiesis Overt-PMF: bone marrow biopsy showing megakaryocytic proliferation and atypia, accompanied by reticulin and/or collagen fibrosis grades 2 or 3	
A2	Clonal abnormalities	Demonstration of JAK2V617F or other clonal marker; in absence of clonal markers, no evidence of secondary bone marrow fibrosis	Presence of JAK2, CALR, or MPL mutation or in the absence of these mutations, presence of another clonal marker^1^ or absence of minor reactive bone marrow reticulin fibrosis	JAK2, CALR, or MPL mutation or presence of another clonal marker^1^ or absence of reactive bone marrow reticulin fibrosis	
A3	Exclusion criteria	Not meeting WHO criteria for the diagnosis of PV, ET, CML, MDS, or other myeloid neoplasm^2^	Not meeting WHO criteria for the diagnosis of PV, ET, CML, MDS, or other myeloid neoplasm^2^	Diagnosis criteria for BCR-ABL1 positive CML, PV, ET, MDS, or other myeloid neoplasms^2^ are not met	
	Minor criterion				
B		a Leukoerythroblastosis/blood erythromyelemia b Increase serum lactate dehydrogenase c Anemia d Palpable splenomegaly	Presence of one or more of the following: a. Anemia no other etiology b. Leukocytosis ≥ 11 × 10^9^/L c. Palpable splenomegaly d. LDH > reference level e. Leukoerythroblastosis	Presence of at least one of three criteria, confirmed by two determinations: a. Anemia not attributed to a comorbid condition b. Leukocytosis ≥ 11 × 10^9^/L c. Palpable splenomegaly d. LDH > reference level	
	Diagnosis:	Diagnosis of PMF requires meeting all 3 major criteria, and at least 2 minor criterion	Diagnosis of PMF requires meeting all 3 major criteria, and at least 1 minor criterion	Diagnosis of PMF requires meeting all 3 major criteria, and at least 1 minor criterion	

*(M)*, male; *(F)*, female; *LDH*, lactate dehydrogenase; *MDS*, myelodysplastic syndrome

^1^If PMF “triple negative” finding another clonal marker must be performed (most common mutations ASXL1, DNMT3A, EZH2, TET2, IDH1/IDH2, SRSF2, SF3B1)

^2^Eliminate secondary myelofibrosis (infection, autoimmune disease, chronic inflammatory disease, lymphoid hematological, metastatic cancer, toxicity)

### Chronic myeloid leukemia

Chronic myeloid leukemia (CML) is a chronic bone marrow disease characterized by extensive granulocyte growth, independent of ligand binding. In blood tests, this is reflected by an increase in the number of granulocytes and blast cells [21]. The hallmark of CML is the t(9;22) (q34;q11.2) transcript, known as the Philadelphia chromosome (BCR-ABL1), which results in the formation of a breakpoint cluster region-tyrosine protein kinase fusion gene. This translocation is responsible for the production of the fusion gene, BCR-ABL1, which encodes a constitutionally active tyrosine kinase that induces cell proliferation. The position of the breakpoint is very irregular, although the most recurrent recombination, m-BCR, M-BCR, and µ-BCR, leads to the formation of a hybrid protein BCR-ABL1 of 190 kDa, 210 kDa, and 230 kDa, respectively [22]. The worldwide incidence of CML ranges from 0.6 to 2.8 cases per 100,000 individuals per year, with variations observed in different regions. For instance, in Egypt, the incidence rate is 0.7 cases per 100,000 individuals, while in Australia and France, it is respectively 1.8 and 1.7 cases per 100,000 individuals [23]. CML diagnosis is established based on a physical examination of the spleen and liver size, a standard biochemical profile including hepatitis B serology, cholesterol, lipase, and hemoglobin A1c values, and an electrocardiogram [24]. The performance of additional diagnostic tests and procedures depends on the patient characteristics, medical history, and comorbidities. The recommended methodologies are cytogenetics/FISH and bidirectional Sanger sequencing [24, 25].

### Essential thrombocythemia

In 1934, Emil and Alfred Epstein Godel [26] described hemorrhagic thrombocythemia, which was later renamed essential thrombocythemia (ET). It is a chronic blood disease characterized by predominant proliferation of the megakaryocytic lineage leading to excessive platelet production with a risk of progression into other forms of MPNs or acute leukemia. The worldwide incidence of ET is estimated to be 1.03 against respectively 1.60 and 0.96 per 100,000 inhabitants in Europe and North America [27, 28], and a prevalence of 38–57 new cases/year/100,000 inhabitants [29, 30]. The sex ratio is approximately 2:1, and all ages are likely to be affected; however, two distinct patient profiles emerge: the first occurs around the age of 30 years, and the second is predominant among women with a median age of 60 years [29]. The etiology of ET is characterized by clonality and neoplasia in the presence of abnormalities, most often JAK2 mutations. This somatic alteration leads to platelet overproduction independent of thrombopoietin stimulation, accompanied by thrombotic or hemorrhagic events. Splenomegaly and hepatomegaly are found in 50% and 15% of cases, respectively, and are part of the diagnostic criteria.

### Polycythemia vera

Decades before William Dameshek, Louis Henri Vera described in 1892 polycythemia vera (PV) as a blood disease. PV is characterized by predominant proliferation, but not exclusively in the erythroid lineage, causing an increase in the total mass of globular cells independent of erythropoietin stimulation. It is an idiopathic disease with geographic variation worldwide, with a median age of approximately 60 years (rarely before the age of 40 years). The PV prevalence in Europe ranges between 0.84 and 0.86 per 100,000, while it was recorded at 0.74 per 100,000 in North America [27]. Eastern European Jews are thought to have a higher incidence than other Europeans and Asians, despite a male-to-female ratio of approximately 2:1 in all races and ethnicities [31]. The study of erythroid progenitors in vitro, in the absence of erythropoietin, aids in the diagnosis of the early stages of the disease or when hidden by iron deficiency. JAK2 gene alterations are present in 80% of PV cases and progression to PMF or acute leukemia (AML) is possible.

### Primary myelofibrosis

Primary myelofibrosis (PMF), also called “Myelofibrosis” or “chronic idiopathic myelofibrosis,” is a chronic blood disease first described in 1879 by Gustav Heuck [32]. This is the rarest and most severe BCR-ABL1 negative myeloproliferative neoplasm. PMF is characterized by the progressive deposition of fibrous connective tissues in the bone marrow (reticulin and/or collagenic fibrosis) associated with extramedullary hematopoiesis (myeloid metaplasia), cytopenia, leukocytosis, and a unique shape and abnormal proliferation of megakaryocytic cells [33, 34], all of which are coupled to voluminous and palpable splenomegaly. PMF etiology is still not well known. The causes of death include leukemic progression, which occurs in approximately 20% of the cases. Many patients also die of comorbid conditions, including cardiovascular events and consequences of cytopenia such as infection or hemorrhagic events [35]. The median age is 65–67 years based on the largest studies from the USA, Europe, and Australia, although it is reported to be as high as 76 years in Sweden [36]. With an overall incidence of approximately 0.47 per 100,000 inhabitants [27], PV and ET can evolve into PMF post-PV or post-ET.

### Molecular biology techniques contribution to classic MPN diagnosis establishment and biological monitoring

A differential diagnosis for all cases of suspected MPN should begin with cytomorphological evaluation of the peripheral blood and bone marrow. In cases where discrimination is not supported by clinical parameters, cytogenetic and anatomopathological data can contribute to establishing diagnosis as well as molecular biomarker detection. The investigation of genetic variants of JAK2, CALR, and MPL has established and integrated features of the diagnostic criteria for MPN since the 2008 revision of the WHO classification (Table 1) of myeloid neoplasms [16, 38]. Recent findings indicate that the Janus kinase–signal transducer and activator of transcription (JAK-STAT) pathway is activated in all MPNs, regardless of the specific driver mutations that are present. Nevertheless, these mutations probably result in additional irregularities in other metabolic pathways. The WHO classification criteria include the status of JAK2 mutations (JAK2V617F and JAK2 exon 12) as major diagnostic criteria for PV, and JAK2, CALR, and MPL mutations in ET and PMF. For a long time, driver mutations were considered mutually exclusive until Mansier et al. [39] revealed that CALR or MPL mutations can coexist in rare “doubly mutated” patients with a low JAK2V617F mutation allele burden [29, 39]. However, the significance of multiple mutations remains unclear.

The challenge of simpler workflow in molecular biology has enhanced the development of new automated polymerase chain reaction (PCR) molecular tools. As the diagnostic value of MPN driver mutations JAK2, CALR, and MPL is crucial, the selection, implementation, and ongoing evaluation of the appropriate laboratory methodology depend not only on the requested mutation but also on the sensitivity and specificity of the technique. The assay’s high sensitivity is critical, as is its rapid performance in screening for MPNs. Indeed, a fraction of hematopoietic clones and their isolated lineages are likely to be mutated, and the risk of progression to more aggressive forms is significant if not managed. In 2023, the WHO recommended the utilization of highly sensitive tests for JAK2V617F, CALR, and MPL, with sensitivity levels of less than 1%, 1–3%, and 1–3%, respectively, in negative cases [40]. The selection of an appropriate technique is crucial for achieving accurate results. Over the last few decades, several tests based on molecular biology and next-generation sequencing (NGS) techniques have been developed, each with its own limitations and advantages. Here, we summarize the most commonly used methods and discuss their relative relevance for each application in classic MPN diagnosis.

### Philadelphia chromosome

The SH1 tyrosine kinase region is the most studied BCR-ABL1 domain owing to its inherent role in CML pathogenesis. The oncogene BCR-ABL1 is indicative of CML, and its identification is commonly performed by karyotyping, which remains the “gold standard” technique [3]. The 210-kDa BCR-ABL1 protein observed in CML retains the Ser/Thr kinase, Rho/GEF, and dimerization (coiled-coil) domains of BCR, fused to the SH, proline-rich (PxxP), deoxyribonucleic acid (DNA) and actin-binding domains. The nuclear localization and entry signals of ABL1 are also conserved. Although BCR-ABL1 contains the majority of the ABL1 gene, it lacks the sequence encoding the N-terminal myristoylation site of ABL1 [41]. In other words, the loss of a portion of the ABL1 gene is responsible for the constitutive activation of BCR-ABL1 kinase.

Detection of the BCR-ABL1 gene has undergone a series of developments, from cytogenetics and immunology to molecular biology. The most common technique used for t(9;22) detection is cytogenetics, in which standard karyotype analysis is performed using peripheral blood or bone marrow samples. Conventional cytogenetic methods analyze the structure and number of chromosomes to identify chromosome abnormalities. Chromosomes are colored using standard R- or G-banding with trypsin Giemsa-stained metaphases [42] and classified using the International System for Cytogenetic Nomenclature Criteria [43]. Cytogenetics is still useful for follow-up as leukemic transformation is characterized by clonal evolution to more complex karyotypes. Immunological methods are used to detect oncoproteins encoded by the fusion gene in peripheral blood (PB) samples, based on antigen–antibody binding reactions. The labeled antibodies bind to fusion proteins and the signals are detected by colorimetry or flow cytometry to identify the presence of fusion proteins [44]. In practice, the limit of detection of CML cells depends on which probes are used, the size of the nucleus, the precise portion of the breakpoint within the ABL1 gene, and the criteria used to define co-localization [45].

BCR-ABL1 fusion genes and proteins present in CML can also be detected using fluorescent in situ hybridization (FISH) [46] or qualitative/qualitative reverse transcriptase polymerase chain reaction (RT-PCR) [24]. FISH is a highly complex and time-consuming process that is also known for its significant financial costs. Furthermore, FISH is not well standardized across laboratories and is a relatively insensitive technique compared to RT-PCR. RT-PCR is capable of detecting a single BCR-ABL1-positive cell in a sample of 100–1000 normal cells, using just 1 μg of RNA [45, 47]. Currently, RT-PCR is the most valuable method for detecting and monitoring BCR-ABL1 expression, although real-time RT-PCR (RT-qPCR) is more sensitive than conventional RT-PCR. dPCR can be more sensitive than RT-qPCR. According to Alikian et al. [48], dPCR is the most accurate method for monitoring CML due to its ability to detect extremely low concentrations of target molecules with high precision, without requiring a standard. Therefore, this approach requires validation. Standardizing assays is crucial, even though a calibration curve may not be essential to quantify target molecules.

Other molecular approaches for direct quantitative detection, such as NGS, duplex sequencing, and digital PCR, can accurately measure BCR-ABL1 transcript levels and monitor CML [49]. Considered as third-generation quantitative techniques, these methods are alternatives to the quantification of molecular diseases that provide more sensitive and specific detection of low levels of disease. Compared with conventional Sanger sequencing, which has a sensitivity Limit of 10–20%, NGS has shown sensitivities in the range of 1–3% (Table 2), with a detection threshold Limited to approximately 1%, mainly due to errors generated by the NGS pipeline [50]. It is interesting to note that the BCR-ABL1 transcript can also be detected by highly sensitive PCR methods in healthy individuals without CML symptoms, thus necessitating a detection cut-off [51]. The measurement of BCR-ABL1 transcript levels using quantitative techniques allows monitoring of the initial response to therapy and the prediction of treatment failure and/or disease progression with more accuracy than karyotyping [52]. Furthermore, in an era in which deep molecular response (DMR) is becoming the target and represents the mandatory prerequisite for a treatment-free remission approach, a highly sensitive and accurate technique for BCR–ABL1 levels monitoring is necessary [53]. With this in mind, digital droplet PCR (ddPCR) and RT-qPCR are excellent alternatives with several practical applications.

**Table 2:** BCR-ABL1 detection methods analytic sensitivity, benefits, and critical points

Technique	Karyotype	FISH	Digital PCR	RT-qPCR	HRM	Droplet Digital PCR (ddPCR)	Sanger sequencing	NGS	Reference
BCR-ABL1 Sensitivity	5–10%	1–5%	3–5%	0.01–10%	1.5–5%	0.001–1%	10–20%	1–3%	[43–52, 54, 55]
Benefits	The gold standard; can detect other structural chromosomal changes, suggesting advanced phase disease	High-sensitivity qualitative detection technique	Achieves absolute quantification of the amplification; no need for calibration curves; lower technical variation; high performance in terms of sensitivity; no need to perform calculation	Very sensitive quantitative PCR	Simple to perform; high speed	High sensitivity; achieves absolute quantification of the amplification of interest without a standard curve	Semi-quantitative; mutation characterization	High sensitivity; low input of DNA/RNA; Detection of known and unknown mutations simultaneous screening of multiple genes in multiple samples	
Critical point	Lowest sensitivity; time-consuming; can only detect large chromosomal changes; requires dividing cells and specialist technician	Relatively insensitive; highly specific to targeted region and will miss other chromosomal changes	No simultaneous screening of multiple genes in multiple samples	Not well standardized across laboratories. more susceptible to contamination or RNA degradation issues	Optimal function requires small amplicon size	High costs; need specific knowledge; absence of standardization	Lowest sensitivity	Validation studies required; high-complexity workflow and analyzing results; intensive labor	

### JAK2V617F mutation

One of the most frequent mutations found in BCR-ABL1-negative myeloproliferative neoplasms is the substitution of valine into phenylalanine at position 617 (V617F) in the JH2 domain of Janus kinase 2. JAK2 encoding for a tyrosine kinase receptor. JAK2V617F mutation is present in approximately 95% of MPN cases. This mutation negates the inhibitory function of the JH2 domain on the kinase activity of the protein, resulting in constitutive activation of the JAK2 signaling pathway [15]. Janus kinase 2 protein (JAK2) is a cytosolic tyrosine kinase of 1132 amino acids encoded by a gene located on the short arm of chromosome 9 (9p24). The point mutation JAK2V617F is responsible for the activation of downstream signaling pathways and mediators of the JAK/STAT pathway via the mitogen-activated protein kinase (MAPK), phosphatidylinositol inositol3 kinase (PI3K), and mechanistic target of rapamycin (mTOR) pathways [58]. Since the discovery of the JAK2V617F mutation in 2005, a plethora of techniques have been developed to target this mutation in MPNs. Two main criteria are important in the test choice: specificity and sensitivity, with a cutoff of mutated allele detection between 1 and 3%. This threshold has been shown to be pathogenically relevant and of clinical significance [56, 57]. According to the “European LeukemiaNet,” the most reliable and sensitive detection technique for JAK2V617F is real-time allele-specific PCR (AS-qPCR). Based on allele-specific PCR (AS-PCR), AS-qPCR is a highly sensitive method with a sensitivity of approximately 0.001–1% and a specificity of 100% [59]. In the last few years, amplification-refractory mutation system (ARMS-PCR) [60], digital PCR (dPCR) [61, 62], and Sanger sequencing have made it possible to obtain absolute quantification of DNA without needing a standard. These techniques are advantageous as they can detect potentially pathogenic variants. Another technique for the detection and quantification of JAK2V617F is aCGH (high-resolution DNA chips) which can also detect novel mutations in JAK2 [61, 63].

In line with all chronic diseases, monitoring the JAK2V617F residual charge remains crucial in the follow-up of the disease. As most JAK2V617F studies conducted using direct matrix sequencing of PCR-amplified genomic DNA are unable to provide the sensitivity required for diagnosis, their routine testing relevance is Limited. Techniques with a sensitivity range of 3–5% may prove insufficient for detecting low allele burden during disease follow-up. Ono et al. [64] have pointed out the usefulness of direct sequencing and the advantage of more sensitive PCR methods in the detection of residual mutation. Other techniques less expensive and more effective like restriction fragment length polymorphism (RFLP-PCR), AS/ARMS-PCR, denaturing high-pressure liquid chromatography (dHPLC), or high-resolution melting PCR (HRM-PCR) seem to be more accurate to achieve this problematique. Several authors have generated ARMS/AS-PCR primer sets for JAK2 c.1849 G > T detection. In addition to representing the gold standard technique for JAK2V617F, ARMS-PCR and AS-PCR have the advantage of a higher apparent sensitivity to small amounts of mutant DNA in a wild-type background (up to 0.1–10% and 1–3% respectively) [60]. Recently, Moradabadi et al. [65] presented a modified RFLP-PCR method. This technique includes the presence of internal controls to verify enzymatic cleavage performance, which reduces the risk of false-positive results and is cost-effective and affordable compared to Sanger sequencing or NGS methods. In 2019, a new detection technique, peptide nucleic acid clamping (PNA-PCR), was developed based on the competition between a PNA probe and primer to bind the same DNA sequence, exploiting the ability of PNA to hybridize to DNA and suppress amplification [66]. This method can be used for single-nucleotide polymorphism (SNP) detection with good sensitivity when the PCR assay uses target-specific probes that bind to mutant sequences. Much more sensitive than sequencing, HRM-PCR can achieve detection thresholds down to 0.5–10% of mutated alleles (Table 3). This is particularly useful for post-medullary allogeneic transplant follow-up, especially in PMF, as well as therapeutic follow-up based on quantification of residual JAK2V617F mutants [67].

**Table 3:** JAK2V617F detection methods analytic sensitivity, benefits, and critical points

Technique	AS-qPCR	RFLP-PCR	dHPLC	ARMS PCR	ASO-PCR	Droplet digital PCR (ddPCR)	qPCR	HRM	Sanger sequencing	NGS	Reference
JAK2V617F sensitivity	0.001–1%	2–10%	1–2.5%	0.1–10%	1.5–5%	0.008–1%	0.01–1%	0.5–10%	10–20%	1–2%	[25, 59, 60, 62, 63, 65, 69–74]
Benefits	High sensitivity; quantitative; simple, fast; allows demonstration of all three genotypes	Convenient and simple	High-throughput; fast	Simple, fast; allows demonstration of all three genotypes	Sensitivity; ideal for large-scale screening	High sensitivity and specificity quantitative detection technique; Simultaneously detects and quantitates mutated allele burden; no need for standard curve	High-sensitivity quantitative detection technique; high specificity, and feasibility	Semi-quantitative detection; simple to perform	Identify known and unknown variant	Identify known and unknown variants in multiple genes and samples at the same time screening	
Critical point	Can only detect target mutations	Time-consuming; operator-dependent; incomplete digestion with the restriction enzyme can cause false-positive results	Post-PCR treatment necessary; technically challenging	Requires post-amplification manipulation; can only detect homozygous and heterozygous	Requires prior knowledge of mutations; ^a^False positives issue	Detection of only targeted variants; moderate cost	Requires two subsequent PCR rounds; detection of only targeted variants; moderate cost	Moderate to low sensitivity; genotyping required association with Sanger sequencing; less reliable at lower mutational levels	Limited by low sensitivity; generates a high percentage of false-negative results	Complexity of data analysis and result interpretation; need to be confirmed by other molecular biology techniques; high cost	

^a^Laboratories may choose to implement a specific threshold to attain 100% specificity, but this approach will inevitably lead to numerous false-negative results

In general, quantitative PCR methods are preferred because of their potential value in measuring the mutant allele burden and monitoring treatment responses. AS-qPCR, ARMS-PCR, droplet digital PCR (ddPCR), and HRM-PCR are within the sensitivity range recommended for the detection of JAK2 exon 14 (V617F) mutation [68].

### JAK2 exon 12 mutation

With the assumption that the JAK/STAT pathway is a major crossroad in BCR-ABL1 negative MPN malfunctions, Scott et al. [8] sequenced the whole JAK2 gene. From a JAK2V617F PV granulocyte-negative patient, they identified a myriad of changes in exon 12. JAK2 exon 12 mutations result in a gain of function, leading to the expression of an erythrocyte phenotype that can evolve towards a transformation into myelofibrosis, unlike those of exon 14. Mutations in JAK2 exon 12 are a characteristic feature of PV, and are exclusively observed in younger patients with hyperplasia of the erythroid lineage with low serum erythropoietin levels, and a negative JAK2V617F profile. JAK2 exon 12 mutations consist of a variety of mutations that can range from substitutions, insertion–deletions (In-Dels), to duplications in the 44 nucleotide portion of JAK2 exon 12, which includes amino acids 533 to 547 of the protein. Small deletions in the reading frame, which occur in approximately 3–12 nucleotides, are the most frequent mutations. These mutations included N542-E543delE (23–30%), E543-D544del, F537-K539delinsL (10–14%), H538-K539delinsL, and R541-E543delinsK (less than 10%) [56]. Over the last decade, a plethora of mutations have been identified. Pietra et al. [70] reported novel duplications in two PV patients involving the substitution F547 (V536-I546dup11, F537-I546dup10 + F547L) [75].

With more than 60 different mutations identified in this region, JAK2 exon 12 mutations exhibit diversity and heterogeneity, making sequencing the most appropriate characterization method. Therefore, this approach’s inherent shortcomings and flaws, the rarity and heterogeneity of mutations described in this region, justify its use for the precise characterization of the variant (Table 4). Strategies for detecting JAK2 exon 12 mutations must possess a considerable degree of analytical sensitivity and the capacity to identify a broad array of nucleotide alterations. Although direct sequencing continues to serve as the gold standard for identifying JAK exon 12 mutations, its limitations include its low sensitivity, which is often insufficient due to low disease prevalence. Other more sensitive techniques have been developed, including high-resolution melting analysis (HRMA), HRM, and dHPLC [63]. Furtado LV et al. [76] described a multiplex fragment analysis-based method that is substitutable with HMR for JAK2 exon 12 screening, as it is a simple, robust, and highly sensitive solution. Therefore, characterization of the mutation cannot get rid of Sanger sequencing. To overcome this obstacle, NGS can be applied as it facilitates the identification of low frequencies of mutant alleles. In summary, the methodology choice depends on the application, and there is no standard detection method for these mutations [77]. Although direct sequencing is less sensitive than high-resolution fusion analysis, which can detect moderate levels of exon 12 mutations in granulocytes, it remains the most commonly used technique because of its ability to detect novel mutations. Despite these limitations, direct sequencing is still a valuable tool for identifying JAK2 exon 12 mutations [78].

**Table 4:** JAK2 exon 12 detection method analytic sensitivity, benefits, and critical points

Technique	AS-qPCR	Locked nucleic acid (LNA)-clamped fragment	Fragment analysis PCR	qPCR	HRM/HRMA	Sanger sequencing	NGS	Reference
JAK2 exon 12 Sensitivity	0.05–1%	0.1–2%	5–10%	–2%	5–10%	10–20%	1.25–2%	[25, 76, 79–83]
Benefits	Highly sensitive; quantitative detection technique	Sensitive; fast; better analytic sensitivity for single bp^1^ substitutions	Simple; robust; highly sensitive; less labor intensive; fast; inexpensive	Highly sensitive; quantitative; suitable for low allele burden detection; can detect multiple mutations at the same time	Simple, reliable, highly sensitive; detect unknown mutations; convenient for large-scale routine screening	Low sensitivity; identify known and unknown variants; high input of sample	Sensitive; robust; allows detection of low allele burden	
Critical point	Detect known mutations only one per assay	Unable to detect substitution mutations and large duplications; generation of pseudo-mutations	Not quantitative	Need to be associated to sequencing; risk of false positives	Variable sensitivity depending on the mutation detected and template quality	False negative issue	Complexity of data analysis and result interpretation; false negative issue; high cost	

^1^* bp*, base pair

### CALR mutations

In 2013, Klampfl et al. [9] and Nangalia et al. [78] discovered mutations in the calreticulin (CALR) gene in BCR-ABL1-negative MPN by sequencing the exome. Mutations in BCR-ABL1-negative MPNs associated with CALR are usually heterozygous and are found in 70% and 84% of ET and PMF cases, respectively [84]. CALR mutations are mainly insertions or deletions in exon 9, which causes a frameshift that results after transcription in a truncated protein devoid of the KDEL recovery sequence. The deletion of 52 base pairs (80–90%) denoted CALRdel52/type I, c.1092_1143del (L367 fs * 46), and the insertion of five base pairs denoted CALR CALRins5/type II (c1154_1155insTTGTC; K385 * 47), in CALR exon 9, are responsible for a decrease in the affinity of the binding sites of the protein. Although both result in mutant proteins, they do not have the same functions. Methods for screening CALR exon 9 mutations must have a minimum sensitivity of 10%, and some of these tests must be used in tandem with Sanger sequencing to provide accurate genotyping. The current reference techniques for CALR mutation detection are PCR fragment analysis or Sanger sequencing, which may substitute for NGS (detection of CALR with a sensitivity of 1–2%) or PCR coupled with bidirectional sequencing [85]. These tools are highly sensitive, require complex work flour, and generate complex data that must be confirmed by alternative molecular genetic methods. Furthermore, despite its relatively high sensitivity (5–10%) for the detection of CALR exon 9 mutations, fragment analysis test is limited by its inability to distinguish the point mutation from the wild-type sequence. HRMA is a PCR-based technique for detecting gene mutations and polymorphisms. This method uses a closed-tube system and examines DNA melting patterns to identify changes in the DNA sequence. Due to its rapidity, sensitivity (2.5–3% for CALR type 1 and 1.25% type 2 mutants, respectively), reliability, and low cost, HRMA represents an effective method for the detection of CALR mutations [86, 87]. HRM is the least robust technique, as it tends to generate false positives, whereas PCR fragment analysis is the simplest method to implement [54]. Nonetheless, HRM and its variant HRMA are more appropriate and sensitive methods than Sanger sequencing (10–20%) for CALR mutation screening (Table 5), both in clinical and research settings. Although Sanger sequencing is complementary and necessary to determine the exact genotypes, the pattern of melting curves does not correlate with the specific CALR mutation types.

**Table 5:** CALR exon 9 detection method analytic sensitivity, benefits, and critical points

Technique	AS-qPCR	Fragment analysis	^a^PNA PCR clamping	HRM/HRMA	CSGE	dHPLC	RQ-PCR	Digital PCR	Sanger sequencing	NGS	Reference
CALR sensitivity	0.8–3.6%	5–10%	0.1–10%	1.25–2.5%	NA	1–5%	1–2%	0.1–1.5%	10–20%	1–2%	[9, 11, 52, 66, 69, 70, 86, 87, 92–94]
Benefits	Rapid; sensitive; reliable; low cost	Known and unknown genetic variant detection; qualitative and quantitative; simple to perform; low cost and rapid	High efficiency, specificity and sensitivity; fast, and easily interpretable	Rapid; sensitive; known and unknown genetic variant detection; simple to perform; low cost and rapid	Sensitive; reliable; low cost	High sensitivity and specificity; qualitative detection	High sensitivity; quantitative; Rapid	High sensitivity; quantitative; rapid	Known and unknown genetic variant detection	Known and unknown genetic variant detection; simultaneous screening of multiple genes in multiple samples	
Critical point	False negative issues in cases with low allele burden; need to be associated with other methods for confirmation; can’t detect a single nucleotide substitution or small deletion/insertion	Moderate to low sensitivity.; preferential amplification of shorter amplicons may lead to over- or underestimation of the mutant allele burden; Sanger sequencing is needed for correctly genotype the CALR variant	Qualitative analysis; can only detect the known mutation	Moderate to low sensitivity; not quantitative; Sanger sequencing is needed for correctly genotype the CALR variants	Can only detect single-base changes and small insertions or deletions; may not distinguish between two closely positioned sequence variations	Relatively low sensitivity; Not quantitative; labor-intensive and time-consuming	Detects only target genetic variants; moderate cost	Detects only target genetic variants; moderate cost	Low sensitivity; not quantitative; moderate cost	Technical limitation; complex workflow bioinformatics analysis; time-consuming	

*NA* Not available

^a^PNA clamped real-time PCR with specific hybridization probes and melting curve analysis

The following methods, including fragment length analysis, ddPCR, qPCR, AS-PCR, pyrosequencing, and NGS, can be utilized as discriminatory techniques. Therefore, they can only differentiate between mutated and wild-type alleles without being able to determine the specific type of mutation present in the disease [59]. These techniques can serve as suitable alternatives for the detection of CALR type-1 and type-2 mutations, despite their inability to identify novel mutations that could be present in the CALR gene. On the other hand, the use of NGS as a screening test for identifying CALR mutations is disproportionate. Conformation-sensitive gel electrophoresis (CSGE) as a high-throughput genetic screening strategy allows mutational analysis of CALR with better sensitivity and accuracy than AS-PCR in detecting CALR mutation subtypes or variants. These outcomes are similar to previously reported data, in which CSGE appeared to be comparable to direct sequencing or denaturing high-performance liquid chromatography (dHPLC) in terms of sensitivity and specificity for mutation detection in breast cancer [88, 89].

Quantitative measurement of CALR mutations is useful for disease monitoring to track molecular responses to treatment, similarly to JAK2V617F monitoring. Recently, Rosso et al. described a novel technique to detect CALR mutations based on peptide nucleic acid direct PCR clamping, which can hybridize very specifically to DNA. Patients with wild-type (WT) CALR and CALR type 1 (DEL) or type 2 (ADD) mutations produce bands of different intensities and lengths [11]. This method could be beneficial in laboratories that do not have the necessary equipment for complex analyses, such as sequencing (Sanger or NGS). It provides a more cost-effective and time-saving solution for advanced molecular diagnosis. In 2014, Vannucchi et al. [90] introduced a new approach for stepwise diagnosis of MPN. Instead of beginning with JAK2 mutation analysis, the authors suggest performing anti-CAL2 immunohistochemistry (CAL2IHC) as the initial step. If CAL2IHC is positive, it essentially rules out the presence of JAK2, MPL, and other mutations. This approach offers several advantages including increased efficiency, cost-effectiveness, and time savings for both patients and clinicians. Despite the limited published literature on the utility of anti-CAL2 immunohistochemistry as a diagnostic tool, CAL2IHC should be considered as an option in CALR management [91].

### MPL mutations

Belonging to the family of homodimeric receptor type I cytokine, MPL is a key factor in the growth and survival of megakaryocytes [95]. Pikman et al. [10] sequenced the MPL gene and documented substitutions and insertions in a portion of five amino acids (K/RWQFP) at codon 515 of MPL exon 10. Abnormalities in MPL are responsible for systematic activation of the thrombopoietin receptor (TPO-R). These acquired mutations constitutively activate the thrombopoietin receptor independently of cytokine binding through the JAK-STAT regulatory pathway. Screening for MPL mutations should be performed in individuals with ET or PMF who do not have JAK2 or CALR mutations. Five major alterations, all within exon 10, nucleotide 1544, and affecting 2 amino acids, have been described: W515L and W515K (most common), W515A, W515S, and W515R (rarer) [96]. Method selection for MPL exon 10 mutation screening must include sufficient analytical sensitivity to detect low-allelic-level mutations. As for the JAK2 and CALR genes, several diagnostic tools have been developed, but many of these techniques have certain limitations, especially a loss of sensitivity when the sample has a low allele burden [97]. Various techniques, including DARMS/ARMS, ASO, HRM, and multiplex AS-PCR, exhibit similar or even lower levels of analytical sensitivity, ranging from approximately 1.5 to 5% (Table 6) [97–99], while RQ-PCR in conjunction with high-resolution analysis (qPCR-HRM) or Sanger bidirectional sequencing [100] may offer increased sensitivity ranging from 0.1 to 0.5%. Alternative methods such as bead-based assays with locked nucleic acid-modified probes, pyrosequencing [101], and singleplex AS-qPCR may also provide comparable results. It is recommended that the specific requirements and limitations of each method be considered before selecting the most appropriate approach. According to Arunachalam et al. [99], the cost of sequencing is about twice that of PCR techniques (AS/ASO/singleplex PCR) for MPL mutations, and the turnaround time for the test is also shorter (4 h for PCR versus 2 days on average for sequencing). The difficulty in implementing certain methods in routine testing stems from the combination of a large number of mutations and the typically low mutational frequency observed in MPL. Boyd et al. [102] established an AS-PCR multiplex capable of detecting four of the most frequent mutations in exon 10 of MPL (W515L, W515K, W515A, and S505N) with a specificity of 100% and a sensitivity of 2.5% [98, 102].

**Table 6:** MPL exon 10 detection methods analytic sensitivity, benefits, and critical points

Technique	AS-qPCR	Multiplex AS-PCR	Pyrosequencing	ASO-PCR	DARMS/ARMS-PCR	LNA-modified probes	RQ-PCR	HRM	Sanger sequencing	NGS	Reference
MPL sensitivity	0.1–0.5%	2.5–5%	0.1–3%	1.5%	1–5%	0.1–1%	0.1–5%	2–5%	15%–20%	1%	[4, 25, 59, 95–98, 100, 101, 103, 104]
Benefits	Sensitive; quantitative; less labor-intensive; less time-consuming; relatively inexpensive	Simple; efficient; sensitive; less labor-intensive; shorter processing time; relatively inexpensive	Quantitative technique; rapid; robust	Sensitive; rapid; cost-effective	Sensitive; specific; no need for special instrument; cost-effective	Highly sensitive; rapid; less labor intensive; quantitative; robust; open platform for multiplexing; cost-effective	Highly sensitive; less time-consuming; cost-effective	Can detect all known and unknown/rare	Known and unknown genetic variant detection	High sensitivity; robust; identification of novel variants	
Critical point	Can only detect target mutations	Not quantitative	Can only genotyping	Not quantitate; cannot detect mutations other than the four known mutations	Intensive labor; can only target one mutant	Can only genotyping; relative quantification	Not quantitative	Can only genotyping; not quantitative	Low sensitivity; not quantitative; moderate cost	Technical limitations; complex workflow bioinformatics analysis; time-consuming	

Detecting methods in MPNs can be broadly categorized into two groups: targeted identification of specific mutations using PCR techniques and sequencing of the entire gene or exon through pyrosequencing, Sanger sequencing, or NGS. Techniques, including PCR, can perform both qualitative and quantitative analyses with varying degrees of sensitivity, allowing the tracking of the target sequence only. In the case of variable mutations, the detection of known mutations as well as potential new pathogenic mutations in the gene is more effective with sequencing techniques. However, it is important to mention the low sensitivity of the second diagnostic approach (2–10%) compared to the sequencing technique (0.01–1%).

#### Epigenetic alterations in TET2, DNMT3A, ASXL1, IDH1/2, SUZ12, LNK, RUNX1, CBL, SF3B1, and U2AF35

In addition to BCR-ABL1, JAK2, CALR, and MPL, mutations in other genes that contribute to the disease characteristics have been described. MPNs occur following a second mutational event, the first of which is a somatic mutation, most often in epigenetic genes, leading to clonal hematopoiesis, which is observed in some healthy subjects [17]. Currently, known MPN-associated epigenetic mutations include TET2, ASXL1, IDH1/2, LNK, CBL, IKZF1, and EZH2. With the advent of NGS studies on BCR-ABL1-negative MPNs, new mutational targets, in addition to driver mutations, have been reported. Some of them are particularly relevant for prognosis and response prediction. These include subclonal mutations in spliceosome machinery genes, regulators of chromatin structure and histone modification, and epigenetic regulators of DNA methylation, which are included in new prognostic scores, especially in PMF [55]. Epigenetic molecular biomarkers, some with prognostic significance such as mutations in LNK (SH2B3), CBL, or DNA methylation genes TET2, DNMT3A, and IDH1, and chromatin structure regulation genes EZH2 and ASXL1 are associated with favorable or unfavorable disease progression, in association with mutations in JAK2, CALR, or MPL [71]. Mutations in epigenetic-associated genes are not exclusive to MPN. Epigenetic modifications do not involve changes in the DNA sequence but are reversible modifications that can influence the expression (or silencing) of genes. Loss-of-function mutations, which may chronologically precede or follow the acquisition of driver mutations, contribute to phenotypic variability and the progression to more aggressive myeloid neoplasms. These changes are not permanent and can be altered in response to environmental factors. The TET2, ASXL1, IDH1 (exon 4), and IDH2 mutations were analyzed in patients with suspected BCR-ABL1-negative MPNs mainly by direct sequencing and/or HRM [12, 105, 106]. Direct DNA sequencing remains the most commonly used and comprehensive molecular technique. It is a valuable research tool with the advantage of being able to detect other rare mutations in selected genes known for their involvement in leukemogenesis. Sanger sequencing is known for its sensitivity of approximately 15–20%, while the sensitivity of NGS is about 1% of the allelic frequency. Ross et al. [107] highlight the importance of using NGS for myeloid somatic mutation panel testing. Targeted panels focusing on 35 key genes commonly mutated in myeloid malignancies offer a more efficient and focused approach compared to broader sequencing methods. These panels are proving particularly valuable for identifying mutations in epigenetic regulators. Advanced techniques like methylation-specific PCR (MSP) and whole genome bisulfite sequencing (WGBS) are revealing the intricate interplay between DNA methylation and MPN development, potentially opening new avenues for therapeutic intervention [108]. In parallel, there is growing interest in developing monoclonal antibodies and vaccines to stimulate immune responses against MPNs. Preliminary research suggests the need for further clinical trials investigating next-generation immune checkpoint inhibitors (ICIs). These ICIs are primarily being explored to target novel immune checkpoints such as LAG-3, TIM-3, TIGIT, VISTA, and HHLA2 [109, 110].

These advancements in understanding the genetic landscape of MPNs have led to a deeper focus on specific mutations associated with disease progression and prognosis. Based on current knowledge, it is widely recognized that genetic alterations in ASXL1, EZH2, IDH1, IDH2, TP53, and SRSF2 genes are associated with short survey and increased leukemic transformation risks. We mainly discuss ASXL1 exon (exon 12), CBL (exons 8 and 9), DNMT3A (exons 15 to 23), IDH1 (exon 4), IDH2 (exon 4), JAK2 (exon 14), MPL (exon 10), NF1 (55 exons), SF3B1 (exons 15 and 16), SUZ12 (exons 10–16), or TET2 (exons 3–11) diagnostic techniques.

Mutations of TET2 impair the function of TET enzymes, resulting in reduced levels of 5-hmc. As reported by McPherson et al. [111], TET2 mutations have been observed in 16% PV, 5% ET, and 17% PMF. TET2 is mainly screened using Sanger sequencing or NGS technics. The mutational profile of ASXL1 is highly variable and associated with poor patient survival. In a PMF case study, the majority of ASXL1 mutations were frameshifts, with p.Gly646TrpfsX12 being the most frequent [112]. ASXL1 and SRSF2 mutations have been associated with inferior overall, leukemia-free, or fibrosis-free survival in both PV and PMF. ASXL1 incidence was reported as follows: 20%, 7%, and 4%, respectively in PMF, PV, and ET [113]. ASXL1 and SRSF2 mutations have been associated with inferior overall, leukemia-free, or fibrosis-free survival in both PV and PMF. Researchers have identified DNA deletions within ASXL1 using high-density oligonucleotide microarrays and comparative genomic hybridization arrays [114–117]. Alterations in exon 1 of the transcription factor bound to runt (RUNX1) or SUZ12 can be identified by sequencing or aCGH [98–100]. LNK and CBL mutations have been identified by using polymerase chain reaction (PCR) coupled with sequencing (bidirectional). Mutations in SF3B1 are common in various hematological malignancies. In MPNs, SF3B1 p.Lys700Glu is mutated in approximately 5% of patients with PV and ET, and in 10% of patients with myelofibrosis (PMF) [93]. Appropriate techniques for detecting SF3B1 and U2AF35 include Sanger sequencing and bidirectional sequencing PCR [118, 119].

The implementation of clinical laboratory sequencing is complex, time-consuming, and requires expertise and specialized equipment. NGS reduces time and manipulation by allowing simultaneous detection of multiple variants in different samples at the same time. Specific NGS screening panels for mutations associated with BCR-ABL1-negative MPNs can help detect residual disease and monitor possible leukemic transformations. In a recent investigation, the utilization of a 22-gene-tailored NGS panel demonstrated the potential for identifying both established genetic variants that are linked to MPNs and new variants that may be biologically relevant [4]. Although the widespread use of NGS techniques has led to a significant reduction in exam costs in recent decades, the implementation of this technology remains relatively expensive compared to the cost of reagents for conventional methods. Despite its low sensitivity, Sanger sequencing is still considered a competitive method as it remains the best tool for validating variants detected by NGS. Sanger sequencing can distinguish true variants from NGS artifacts or sequencing bias. However, NGS variant confirmation guidelines should consider alternative methods with higher sensitivity, such as ddPCR or AS-qPCR, followed by HRM. Although these methods have limitations, they may be useful for certain applications.

## Conclusion

In MPNs, truncated proteins and mutations in signaling pathways can have a dominant-negative effect. Since 2005, MPN biology has entered the molecular era without losing its clinical or anatomopathological relevance. As described in this article, molecular diagnosis is essential for the management of MPN patients. It is imperative to provide an integrated state-of-the-art list that prioritizes diagnosis and monitoring tools for these diseases. Most developed countries employ the molecular diagnosis methods discussed here, such as AS-PCR, RT-qPCR, FISH, Sanger sequencing, or NGS. These techniques can enhance patient follow-up and management. In fact, the consensus from the Asian Myeloid Working Group (AMWG) emphasizes that accurate molecular diagnosis not only aids in differentiating MPNs from reactive conditions but also informs treatment decisions, risk stratification, and monitoring of disease progression [120]. By integrating these advanced molecular techniques into clinical practice, healthcare providers can enhance patient outcomes and tailor therapies to individual needs, ultimately improving the quality of life for those affected by MPNs. Therefore, digital PCR, duplex sequencing, real-time sequencing, and NGS molecular diagnosis platforms will take time to be implemented in clinical practice in low/middle-income countries. This underscores the disparity in data available for these diseases worldwide. The necessity for accessible methods that prioritize the requirements of clinicians and populations while remaining efficient cannot be overstated. This is in line with the key objective of this study to identify and evaluate methods for detecting MPN-related genetic abnormalities. Therefore, the choice of technique depends on the target, the qualitative or quantitative purpose of the analysis, and the sensitivity, specificity, and reproducibility of the method. For these reasons, biologists should ensure that appropriate techniques have the following attributes: a low detection limit (less than 1% for diagnosis and at least 0.1% for monitoring minimal residual disease); a specificity of almost 100% (minimizing the level of false positives); and ease of transfer between laboratories, which guarantees the reproducibility of the protocol.

## Data Availability

Data sharing is not applicable to this article as no datasets were generated or analyzed during the current study.

## Abbreviations

ADD: Addition
ARMS: Amplification refractory mutation system
AS-PCR: Allele-specific polymerase chain reaction
ASO-PCR: Allele-specific oligonucleotide polymerase chain reaction
BCR-ABL: Breakpoint cluster region – Abelson leukemia
CAL2IHC: Anti-CAL2 immunohistochemistry
CSGE: Conformation-sensitive gel electrophoresis
CML: Chronic myeloid leukemia
DARMS: Dual amplification refractory mutation system
DNA: Deoxyribonucleic acid
DEL: Deletion
dHPLC: Denaturing high-performance liquid chromatography
ddPCR: Digital droplet polymerase chain reaction
ET: Essential thrombocythemia
FISH: Fluorescence in situ hybridization
HRM: High-resolution melting
HSCs: Hematopoietic stem cells
JH2: Janus kinase 2
KDa: Kilodalton
LNA: Lock nucleic acid
MAPK: Mitogen-activated protein kinase
MPN: Myeloproliferative neoplasm
mTOR: Mammalian target of rapamycin
NGS: Next-generation sequencing
PIP3K: Phosphatidylinositol inositol3 kinase
PNA: Peptide nucleic acid
PCR: Polymerase chain reaction
PCR–RFLP: Polymerase chain reaction–restriction fragment length polymorphism
PMF: Primary myelofibrosis
PB: Peripheral blood
PV: Polycythemia vera
qPCR: Quantitative polymerase chain reaction
RQ-PCR: Quantitative reverse transcriptase polymerase chain reaction
RT-PCR: Reverse transcriptase polymerase chain reaction
SNP: Single nucleotide polymorphism
SSCP: Single-strand conformational polymorphism
TPO/R: Thrombopoietin receptor
WHO: World Health Organization
WT: Wild-type
